# Dynamics of conflict during the Ebola outbreak in the Democratic Republic of the Congo 2018–2019

**DOI:** 10.1186/s12916-020-01574-1

**Published:** 2020-04-27

**Authors:** Moritz U. G. Kraemer, David M. Pigott, Sarah C. Hill, Samantha Vanderslott, Robert C. Reiner, Stephanie Stasse, John S. Brownstein, Bernardo Gutierrez, Francis Dennig, Simon I. Hay, G. R. William Wint, Oliver G. Pybus, Marcia C. Castro, Patrick Vinck, Phuong N. Pham, Eric J. Nilles, Simon Cauchemez

**Affiliations:** 1 Department of Zoology, University of Oxford, Oxford, UK; 2 Harvard Medical School, Harvard University, Boston, USA; 3 Computational Epidemiology Group, Boston Children’s Hospital, Boston, USA; 4 Institute for Health Metrics and Evaluation, University of Washington, Seattle, USA; 5 Department of Health Metrics Sciences, School of Medicine, University of Washington, Seattle, WA USA; 6 Oxford Vaccine Group & Oxford Martin School, University of Oxford, Oxford, UK; 7 European Union Delegation to the Democratic Republic of the Congo, Kinshasa, Democratic Republic of the Congo; 8 School of Biological and Environmental Sciences, Universidad San Francisco de Quito USFQ, Quito, Ecuador; 9 Yale-NUS College, Singapore, Singapore; 10 Environmental Research Group Oxford, Department of Zoology, University of Oxford, Oxford, UK; 11 Department of Global Health and Population, Harvard T.H. Chan School of Public Health, Boston, USA; 12 Program on Infectious Diseases and Emergencies, Harvard Humanitarian Initiative, Harvard University, Cambridge, USA; 13 Brigham and Women’s Hospital, Boston, USA; 14 Mathematical Modelling of Infectious Diseases Unit, Institut Pasteur, CNRS, UMR2000, Paris, France

**Keywords:** Ebola, Conflict, Violence, Democratic Republic of the Congo, Outbreak

## Abstract

**Background:**

The 2018–2019 Ebola virus disease (EVD) outbreak in North Kivu and Ituri provinces in the Democratic Republic of the Congo (DRC) is the largest ever recorded in the DRC. It has been declared a Public Health Emergency of International Concern. The outbreak emerged in a region of chronic conflict and insecurity, and directed attacks against health care workers may have interfered with disease response activities. Our study characterizes and quantifies the broader conflict dynamics over the course of the outbreak by pairing epidemiological and all available spatial conflict data.

**Methods:**

We build a set of conflict variables by mapping the spatial locations of all conflict events and their associated deaths in each of the affected health zones in North Kivu and Ituri, eastern DRC, before and during the outbreak. Using these data, we compare patterns of conflict before and during the outbreak in affected health zones and those not affected. We then test whether conflict is correlated with increased EVD transmission at the health zone level.

**Findings:**

The incidence of conflict events per capita is ~ 600 times more likely in Ituri and North Kivu than for the rest of the DRC. We identified 15 time periods of substantial uninterrupted transmission across 11 health zones and a total of 120 bi-weeks. We do not find significant short-term associations between the bi-week reproduction numbers and the number of conflicts. However, we do find that the incidence of conflict per capita was correlated with the incidence of EVD per capita at the health zone level for the entire outbreak (Pearson’s *r* = 0.33, 95% CI 0.05–0.57). In the two provinces, the monthly number of conflict events also increased by a factor of 2.7 in Ebola-affected health zones (*p* value < 0.05) compared to 2.0 where no transmission was reported and 1.3 in the rest of the DRC, in the period between February 2019 and July 2019.

**Conclusion:**

We characterized the association between variables documenting broad conflict levels and EVD transmission. Such assessment is important to understand if and how such conflict variables could be used to inform the outbreak response. We found that while these variables can help characterize long-term challenges and susceptibilities of the different regions they provide little insight on the short-term dynamics of EVD transmission.

## Background

On August 1, 2018, the Democratic Republic of the Congo (DRC) declared an Ebola (EVD) outbreak in the eastern part of the country. Almost 1 year later, on July 17, the World Health Organization (WHO) declared the outbreak in the DRC a Public Health Emergency of International Concern (PHEIC) [1]. By then, the outbreak had grown to become the second largest ever recorded, largely concentrated in the eastern provinces of North Kivu and Ituri, with 3145 notified cases of which 3034 were confirmed. Among them, 2098 people have died.

Successful containment requires important efforts to detect cases, conduct thorough follow-up investigations, monitor case contacts, implement ring vaccination with established vaccines, and rapidly isolate patients with symptoms [2]. Much focus has been on the role of directed attacks on Ebola treatment centers (ETCs) at the country level and how they have impacted the efficacy of vaccination campaigns and the potential delay of isolation of symptomatic patients [3]. For example, conflicts and direct attacks on health workers have resulted in the temporary and permanent suspension of Ebola treatment centers in six locations including Butembo and Katwa [4].

The response to the current outbreak, however, is also complicated by local distrust and history of conflict (unrelated to the outbreak) [5–7]. The confluence of pre-existing violence, mistrust, and political instability has resulted in the outbreak continuing to spread geographically and intensifying in affected regions despite ongoing response efforts. Disrupting the movement of individuals and response workers, conflicts impede surveillance and delay or interrupt response activities in some communities [7]. Similarly, individuals flee areas of violence in unpredictable ways which may increase the risk of geographic spread of disease and impacts delivery of basic health care services including vaccines. The Ebola outbreak may also be instrumentalized to fuel conflict and therefore exacerbates its negative indirect effects that have yet to be assessed.

Focus to date has been on the direct relationship between Ebola-related attacks and disease transmission, but the broader context of conflict in the region is less clear. Here, we aim to address this gap. We use exclusively openly available data to characterize the dynamics of conflict and how it has changed over the period of the outbreak. Further, we use geographically precise conflict and epidemiological data to understand the association between conflicts that are not directly related to the EVD outbreak and EVD transmission dynamics.

## Methods

### Study setting

**Fig. 1:**
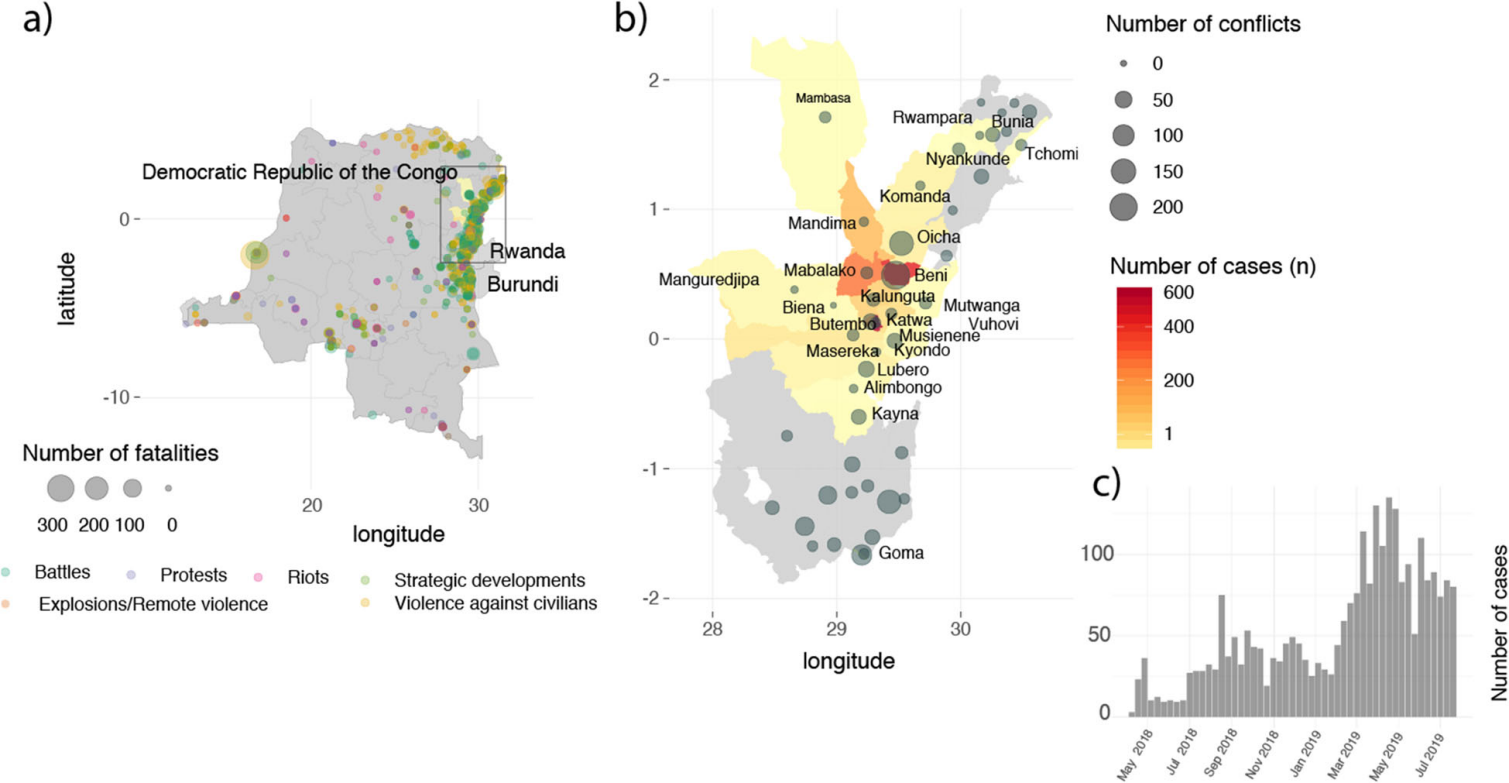
**a** Map of the study areas of the Democratic Republic of the Congo (DRC) and health zones in the provinces of North Kivu and Ituri (box). Dots represent the precise location of conflicts in the DRC during the time of the ongoing outbreak (August 1, 2018, to July 26, 2019). The color of the dots represents the type of conflict, and the size represents the number of deaths. **b** Map of the North Kivu and Ituri provinces. Colors represent the number of EVD cases, and circles represent the number of conflicts during the outbreak (August 1, 2018, to July 26, 2019). **c** Epidemic curve of EVD during the 2018–2019 outbreak

Our analysis is focused on health zones in North Kivu and Ituri provinces in eastern DRC (Fig. 1a). Health zones are the smallest administrative unit for which epidemiological data are available from the DRC MoH (Fig. 1a box, Additional file 1). Epidemiological analyses include only health zones that have reported one or more cases in the current PHEIC.

### Epidemiological data

Epidemiological data were abstracted from daily reports from the Ministry of Health (MoH) of the DRC [8] detailing confirmed and suspected cases at the resolution of the health zones (Additional file 1). We reconstructed daily case data for the time period August 3, 2018, to July 21, 2019, from daily reports of the cumulative number of cases per health zone. We did so by subtracting case counts today from the previous day and did so going back to the start of the outbreak. When the difference was negative, we assumed there were no new cases on that day. Data can be directly downloaded from [9].

### Conflict variables

We combined two databases containing information about conflict events in the DRC.

We assessed pre-outbreak levels of conflict and violence by health zone using the Uppsala Conflict Data Program Georeferenced Events Dataset (UCDP GED) [10]. The UCDP GED is an event dataset that disaggregates three types of organized violence (state-based conflict, non-state conflict, and one-sided violence) both spatially and temporally. The bases for this dataset are publicly reported news (including local radio stations such as Radio Okapi for the DRC), reports from non-governmental organizations, case studies, and historical archives. Each event is defined as an instance of organized violence with at least one fatality and includes additional information such as the date, geographical location (latitude and longitude), and identifiers that allow the dataset to be linked to and merged with other datasets. Conflicts are categorized into battles, explosions/remote violence, protests, riots, strategic developments, and violence against civilians. A detailed description of the data and methodology can be found here [10, 11]. We extracted data from the UCDP GED dataset for 29 years, from January 1, 1989, to July 31, 2018. From this dataset, we constructed a pre-outbreak conflict variable by aggregating numbers of conflicts and deaths at the health zone level.

We assessed levels of conflict and violence over the course of the outbreak and by health zone using the Armed Conflict Location Events Dataset (ACLED). The ACLED data were abstracted from local, regional, national and continental media that were reviewed on a daily basis; reports from NGOs or international organizations used to supplement media reporting; selected social media accounts, including Twitter and Telegram; and information and data provided through partnerships with local conflict observatories in hard-to-access cases. The data were then coded to include information about the type of event (battles, explosions/remote violence, violence against civilians, protests, riots, and strategic developments), geographic information, time of event, and source. A full description of the methodology can be found here [12]. We then aggregated daily conflicts to the health zone level in the DRC using data only from August 1, 2018, to July 26, 2019. Further, we aggregated data for all locations in the DRC that were outside the affected provinces. We created two distinct conflict variables, one which was simply the number of conflicts by category, health zone, and day and a second one that was the number of deaths by health zone and day that are directly related to conflict events. For a sensitivity analysis, we also divided the data into the different conflict types and recorded whether the event was directly related to the Ebola outbreak if the terms “Ebola” or “ebola” were mentioned in the report.

### Spatial covariates

Data on the human population size per health zone for 2019 was extracted from openly available spatial population data available to download here [13]. Data on administrative boundaries in the study region were obtained from a Database of Global Administrative Areas (GADM). We used satellite data to estimate population numbers because the last population census was conducted in 1984 and may therefore be a considerable underestimate of the population.

### Statistical analysis

In each health zone, we identified time periods of substantial uninterrupted EVD transmission defined as no more than 14 days between two consecutive cases and at least 20 cases (below did not yield robust results) in the whole time period and the time period is at least two generation times (> 28 days). There can be multiple time periods of substantial uninterrupted transmission in a given health zone. For each time period of substantial uninterrupted transmission, we reconstructed bi-weekly temporal trends in the instantaneous reproduction number using the EpiEstim package [14], excluding the first 14 days of the time period (i.e., about one generation) as suggested by Cori et al. [14]. The reconstruction was performed under the assumption that the EVD serial interval had a Gamma distribution with mean 15.3 days and standard deviation of 9.3 days [15]. We used vague gamma default priors for reproduction numbers (mean = 5, standard deviation = 5) [14].

To explore the potential association between the different types of conflicts and EVD transmission, we applied univariate linear regression analysis between bi-weekly reproduction numbers and the number of conflicts (battles, episodes of violence against civilians, or number of conflict-associated deaths) during time periods of substantial uninterrupted transmission. We also assessed the association for different time lags from 0 to 4 weeks. We did not use raw case numbers to explore potential associations because of the inherent delay between shifts in transmission and case numbers.

In addition, we performed a regression analysis (using Pearson correlation) between the incidence of conflict by 10,000 per health zone and the incidence of disease by 10,000 which were performed for all 46 health zones for the entire period of the outbreak. All analyses were performed in R version 3.5.1 [16].

## Results

### Conflicts in the pre-outbreak period

In the pre-outbreak period, between 1989 and 2018, 3799 conflicts were reported in the DRC. The outbreak of Ebola in the Eastern DRC is occurring in an area of violence (Fig. 1a, b), including political instability following the presidential elections in December 2018. We found that, in the pre-outbreak period in the year 2017, the incidence of reported conflicts per capita in EVD-affected health zones in North Kivu and Ituri was 630-fold higher than in the remainder of the DRC (Supplementary Appendix, Figure S2).

### Conflicts during the Ebola outbreak

We tabulated 2035 conflict events in the DRC between the declaration of the outbreak on August 1, 2018, and July 26, 2019. The majority of conflict events were classified as battles (33%) and violence against civilians (35%), and these proportions do not vary significantly over the study period (Table 1). Among all recorded conflicts during the study period, 40% included at least one death and 10% caused more than five deaths (Supplementary Appendix). The largest number of deaths from a single event was during an attack on the city of Yumbi in western DRC when 348 deaths were reported ([17], Supplementary Appendix, Figure S1). Protests and riots were more prevalent in regions outside Ituri and North Kivu (Table 1).

**Table 1:** Summary of conflict per geographic area in the Democratic Republic of the Congo between August 1, 2018, and July 26, 2019

	Conflict categories							Incidence	Human population
	Battles	Explosions/remote violence	Protests	Riots	Strategic developments	Violence against civilians	Total	Conflict incidence (per 10,000)	Population
Affected health zones (*n*)^a,b^	224	3	92	33	33	226	611	1.28	4,792,553
Affected health zones (% of number of conflicts)^a,b^	33.58%	50.00%	33.82%	19.88%	16.10%	31.39%	30.01%	–	5.21%
Health zones in North Kivu and Ituri that are adjacent but not affected^a^ (*n*)	161	2	15	12	28	176	394	0.96	4,084,535
Health zones in North Kivu and Ituri that are adjacent but not affected^a^ (% of number of conflicts)	24.14%	33.33%	5.51%	7.23%	13.66%	24.44%	19.35%	–	4.44%
All other areas in the DRC^a,c^ (*n*)	282	1	165	121	144	318	1031	0.12	83,053,912
All other areas in the DRC^a,c^ (% of total number of conflicts)	42.28%	16.67%	60.66%	72.89%	70.24%	44.17%	50.64%	–	90.34%
Total number of conflicts^a^ (n)	667	6	272	166	205	720	2036	0.22	91,931,000
Total number of conflicts (%)	32.76%	0.29%	13.36%	8.15%	10.07%	35.36%	100%	–	100%

^a^Time period of August 1, 2018, to July 26, 2019

^b^Alimbongo, Beni, Biena, Bunie, Goma, Butembo, Kayna, Kyondo, Lubero, Manguredjipa, Masereka, Mutwanga, Oicha, Komanda, Mambasa, Nyankunde, Rwampara, Tchomia, Kalunguta, Katwa, Mabalako, Mandima, Musienene, Vuhovi

^c^The Democratic Republic of the Congo

We tabulated 1004 conflict events (49%) in the outbreak regions, Ituri and North Kivu (Table 1). The number of deaths in the outbreak period (ACLED database) was correlated with that in the pre-outbreak period illustrating that no major shifts in the locations were conflicts were reported occurred (Uppsala database) (Pearson’s *r* = 0.45, 95% CI 0.2–0.67, *p* value < 0.001, Supplementary Appendix, Figure S3 and Figure S5).

### Ebola transmission

**Fig. 2:**
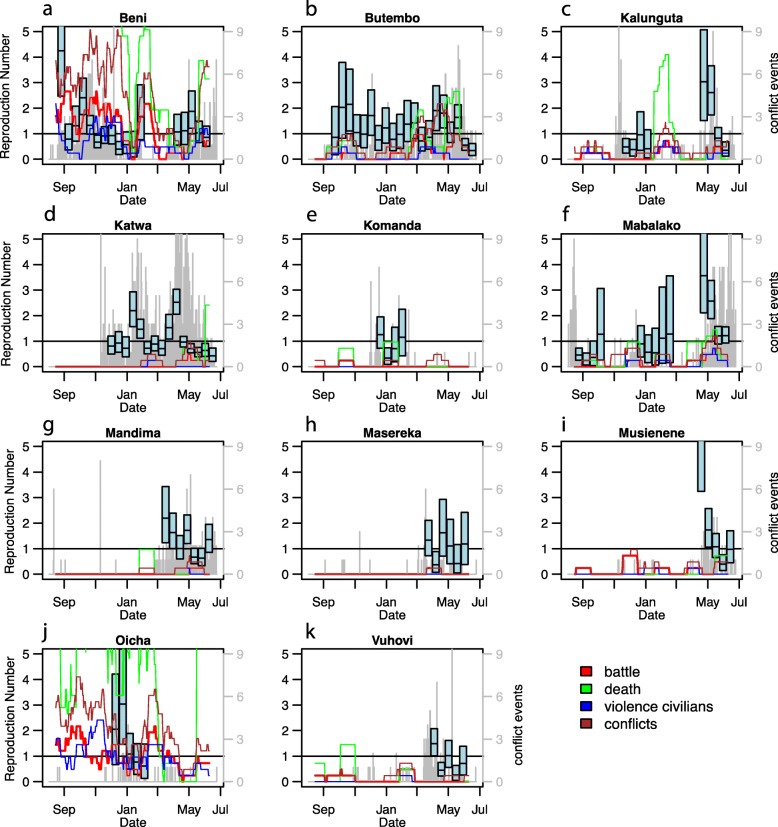
Trends in the bi-weekly reproduction number during time periods of substantial uninterrupted transmission (blue box plot indicating mean and 95% credible interval), the average weekly number of conflicts smoothed over a 1-month time period (colored lines), and the number of cases (gray bars, right hand side axis)

**Fig. 3:**
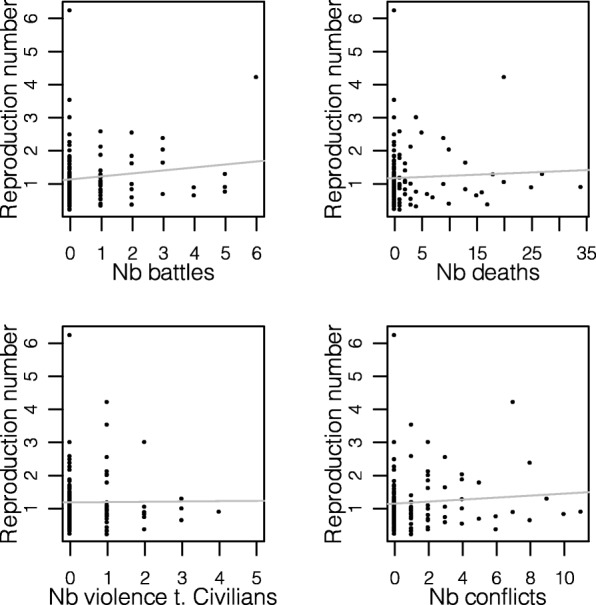
Association between the reproduction number and the number of battles (**a**), deaths (**b**), violence towards civilians (**c**), and conflicts (**d**). The gray lines represent the fitted regression line between the reproduction number and the number of conflicts at a health zone level for periods of sustained, uninterrupted transmission. A full table of correlation coefficients is shown in Table S1

We identified a total of 15 time periods of substantial uninterrupted transmission distributed across 11 health zones and 120 bi-weeks (Fig. 2). These time periods had an average duration of 8 bi-weeks (range: 5–21 bi-weeks) and an average bi-weekly reproduction number of 1.2 (range 0.2–6.3). No significant association was identified between the reproduction number and the number of conflicts during short bi-week intervals of substantial uninterrupted transmission (Fig. 3, Table S1). Further, no significant association was found between the presence of conflict and the presence of disease transmission when accounting for temporal dependence of the EVD case count data.

**Fig. 4:**
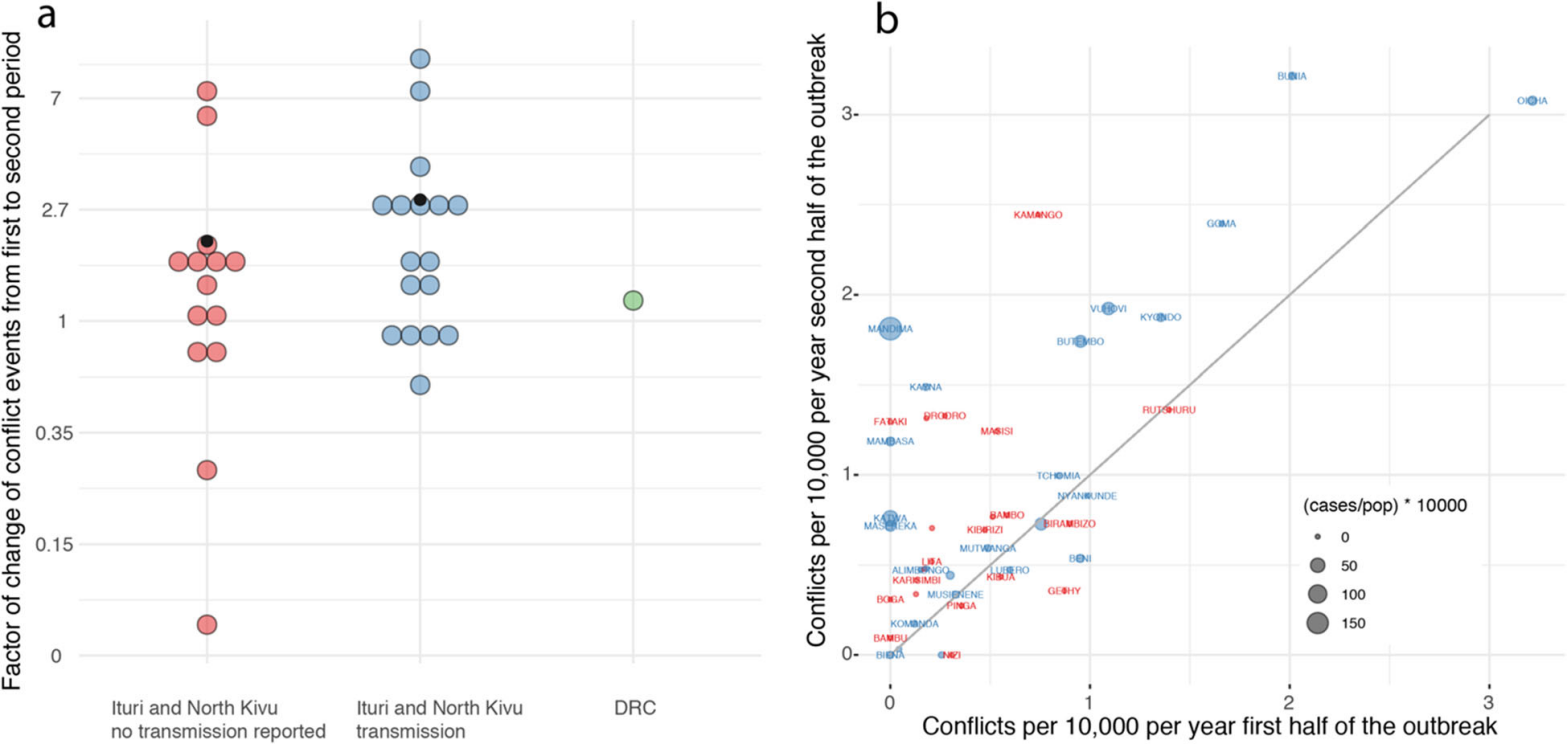
**a** Change between the number of conflicts during the first half of the outbreak (August 1, 2018–January 31, 2019) vs. the second half of the outbreak (February 1, 2019–July 26, 2019) on a log scale (factor change). Blue dots show the areas that reported transmission. Red dots indicate the areas that are in North Kivu and Ituri but did not report transmission. Green is the rest of the Democratic Republic of the Congo. Black dots represent the population-weighted mean of conflicts per group which are 2.7 in health zones that reported transmission, 2.04 in health zones in North Kivu and Ituri that did not report transmission, and 1.32 in the rest of the Democratic Republic of the Congo. Between the initial phase of the outbreak (August 2018–January 2019) and the second phase of the outbreak (February 2019–July 2019), on average, the incidence of conflict events (by day) increased by a factor of 2.75 (Fig. 2a) in health zones that reported transmission (*p* value < 0.05). Health zones in Ituri and North Kivu that did not report transmission were not statistically significantly different (*p* value = 0.05017) but on average experienced a doubling in conflict events (factor 2.04). **b** The relationship between the number of conflicts per 10,000 individuals in the first half of the outbreak (August 2018–January 2019) vs. the second half of the outbreak (February 2019–July 2019). All points that fall above the gray line see an increase in conflict events in the second half of the outbreak. The correlation between the reported incidence of conflict during these two time periods is *R*^2^ = 0.95 (*p* value < 0.01, regression line not shown)

In general, however, we find that over the entire outbreak period there is a weak but significant association between the number of conflicts (considering all conflict types) per 10,000 and the incidence of EVD per 10,000 at the health zone level (Pearson’s *r* = 0.33, 95% CI 0.05–0.57, Figure S6). After an initial decline of the case numbers in December 2018, case numbers started to rise again in February 2019 (Fig. 1d). During that second period, the majority of health zones in North Kivu and Ituri experienced an increase in conflicts. Between the initial phase of the outbreak (August 2018–January 2019) and the second phase of the outbreak (February 2019–July 2019), on average, the incidence of conflict events (by day) increased by a factor of 2.75 (Fig. 4a) in health zones that reported transmission (*p* value < 0.05, *χ*^2^ test). Health zones in Ituri and North Kivu that did not report transmission were not statistically significantly different (*p* value = 0.05017, *χ*^2^ test) but on average experienced a doubling in conflict events (factor 2.04). In the rest of the DRC (excluding Ituri and North Kivu provinces), conflicts increased by a factor of 1.32 (Fig. 4a).

There are subtle differences between health zones. We identify a number of distinct location-conflict characteristics: First, some areas have no incidence of reported conflict prior to the outbreak but experience conflict after cases were reported (Mandima, Katwa, Mambasa, Masereka, Fig. 4b). These locations together account for nearly one third of all cases. Secondly, there are health zones in Ituri and North Kivu that see increases in the number of conflicts but no reported transmission. Thirdly, there are high-conflict health zones such as Beni, Vuhovi, Butembo, Kyondo, Oicha, and Bunia, where there are only slight increases in reported conflict over these two periods (Fig. 4b).

## Discussion

Our study identifies and quantifies trends in conflicts during the current EVD outbreak in eastern DRC. Previous work has focused mostly on the direct impact of Ebola-related violent attacks on EVD transmission [2, 3]. Our aim here was to look at the broader context of violence and incidences of conflict and its association with EVD transmission dynamics.

We found that regions that have large, systematic conflicts may be more vulnerable to large EVD outbreaks although slight changes in reported conflicts did not appear to be associated with short-term variations in EVD transmission potential. This suggests that the different variables (number of conflict events and deaths, stratified by type of event) we used to monitor the broader context of conflicts may be better suited to characterize long-term challenges and susceptibilities in a region than to inform short-term predictions of EVD transmission in that region. However, our result that conflict is associated with the magnitude of the outbreak in each health zone illustrates the potential long-term implications of conflict on public health infrastructure and thus incidence of disease. Interestingly, we also observe that in locations that reported EVD transmission, violence, and conflict, events increased more during the duration of the outbreak than what was observed in other regions. Although our study is observational and therefore can only identify associations, it suggests that the EVD outbreak may further destabilize fragile regions and fuel conflicts. This result is supported by evidence about the mistrust associated with the governments’ decision to ban populations in the affected provinces to participate in the presidential election [6]. Violence and tensions around the period of the elections (increase in violent events in December 2018–January 2019, Fig. 3) and subsequent contested results likely undermined outbreak control efforts.

Even though our results offer an interesting and plausible relationship between the incidence of disease and how it affects conflict, we cannot conclude a causal link between them from such a descriptive study [18]. It has been shown in related work that there is a robust relationship between conflict and disease-related mortality [19, 20]. Importantly, even though we did not find a strong relationship between short-term transmission dynamics of EVD and conflict, the lack of robust and accurate data on conflict and transmission may be one of them. However, we highlight that disease and conflict reporting delays need to be better understood in order to make robust quantitative assessments about the complex relationship between conflict and infectious diseases.

While the specific mode and magnitude of the interactions between conflict events and transmission of infectious diseases is likely to be very localized, our study provides an example of the importance of including general conflict variables in future, systematic studies on types of instability and conflict and their magnitude and epidemiological effect. Such studies will likely be important when planning for eradication of for example polio, malaria, and other infectious diseases.

The shifts of reproduction number estimates from as low as 0.2 to above 1 in periods of uninterrupted transmission are indicative of potential resurgence of EVD in locations due to either re-introduction or localized super-spreading events. An important area for future investigations is to explore how conflicts may affect the spatial spread of EVD for example by increasing the risk of spatial expansion into areas that were previously unaffected [21, 22]. This could be done by monitoring movements out of areas that experience large number of conflicts. Such analyses would help decide where and when to establish surveillance and potentially prioritize vaccine delivery [23]. Integrating conflict variables into outbreak analysis alongside other societal and ecological factors will be essential to better understand the size, duration, and risk of geographic spread in this and future outbreaks [24]. Further, it will be important to include predictive maps of conflict when predicting the spatial and temporal spread of EVD in the future [25].

This study had a number of limitations. First, conflict data are abstracted using a standard protocol but data was in part from online, self-reported sources which have inherent biases. Events that trigger reports are those that are larger and more severe. Those biases are related for example to the level of internet penetration and wealth which has been shown to be biased towards urban areas [11] and variation may be higher for small events compared to large events. We therefore assume that our estimates are biased towards larger population areas (e.g., cities). Second, while an increase in the number of conflicts was observed in all regions, this increase was higher in regions affected by EVD. We cannot rule out the possibility that this was due to increased reporting in these regions. Third, epidemiological data on cases are abstracted from daily reports and may be biased towards larger case clusters. Small clusters may have not been reported which bias estimates of the reproduction number upwards. Other biases include the potential delay in reporting and variation across different spatial locations, especially as data is abstracted from cumulative case reports rather than daily case counts. Genomic sequencing is done at a large scale during the outbreak, and we hope that estimates of the transmission dynamics from genomic analysis can be compared to those presented here [26, 27]. Whether the drop in cases before the presidential election is influenced by reporting will need to be further investigated. We only characterized the reproduction number only in areas (*n* = 15, 64% of all reported cases) that have sufficient numbers of cases to estimate the reproduction number over time. The remaining 36% of cases are reported in 13 other health zones with insufficient data to estimate the reproduction numbers for these health zones. Conflicts are expected to negatively affect reporting, and areas that are the most affected by conflicts may be the ones less likely to report EVD cases which may mask a positive association between conflicts and EVD transmission. For example, cases were reported as early as April 30, 2018, but the outbreak was only declared on July 31, 2018. We did not include potential sensitivities of serial interval distributions that change due to conflict.

## Conclusions

We characterized the association between variables documenting broad conflict levels and EVD transmission. Such assessment is important to understand if and how such conflict variables could be used to inform the outbreak response. We found that while these variables can help characterize long-term challenges and susceptibilities of the different regions they provide little insight on the short-term dynamics of EVD transmission.

## Supplementary information

**Additional file 1 : Figure S1**. Histogram of the number of fatalities per conflict event using data from August 1, 2018 to July 26, 2019. **Figure S2**. Ratio of conflict events during the outbreak vs before the outbreak for health zones in Ituri and North Kivu that did not have transmission, those health zones with transmission and all other regions in the Democratic Republic of the Congo. Data come from two different sources (Uppsala and Armed Conflict Location & Event Data). **Figure S3**. a) Correlation of number of conflicts before the outbreak (1989 – July 31, 2018) and during the outbreak for each of the affected health zones. Gray line shows the regression line and shaded area is 95% confidence interval. b) Correlation between number fatalities before and after the conflict using the same time intervals than in a). Data are shown on the log scale. Gray line shows the regression line and shaded area is 95% confidence interval. **Figure S4**. Number of conflict events in the first half of the outbreak (August 2018 – January 2019) vs. second half of the outbreak (February 2019 – July 2019). Each dot represents one health zone in North Kivu and Ituri. **Figure S5**. Number of conflict events before the outbreak normalized by year (2017) and capita and during the outbreak normalized by year and capita. **Figure S6**. Number of conflicts per 10,000 vs. number of cases per 10,000 (Pearson’s r = 0.33, 95% CI: 0.05–0.57, *p*-value < 0.05). Blue dots represent areas that had reported Ebola transmission. Red dots represent locations that did not report transmission. **Table S1.** Coefficients of the linear univariate regression of the bi-weekly reproduction number (y) and conflict variables (x) (y = a + b*x).

## Data Availability

Data used in this study are openly available. Conflict variables are available from https://ucdp.uu.se/ and https://www.acleddata.com/. Epidemiological data is available through this link: https://docs.google.com/spreadsheets/d/e/2PACX-1vSrr9DRaC2fXzPdmOxLW-egSYtxmEp_RKoYGggt-zOKYXSx4RjPsM4EO19H7OJVX1esTtIoFvlKFWcn/pub?gid=1564028913&single=true&output=csv). Administrative boundary data are available from https://gadm.org/.

## References

[1] World Health Organization (WHO) (2019). Ebola outbreak in the Democratic Republic of the Congo declared a Public Health Emergency of International Concern.

[2] Ilunga Kalenga O, Moeti M, Sparrow A, Nguyen V-K, Lucey D, Ghebreyesus TA (2019). The ongoing Ebola epidemic in the Democratic Republic of Congo, 2018–2019. N Engl J Med.

[3] Wells CR, Pandey A, Ndeffo Mbah ML, Gaüzère B-A, Malvy D, Singer BH, et al. The exacerbation of Ebola outbreaks by conflict in the Democratic Republic of the Congo. Proc Natl Acad Sci. 2019:201913980. 10.1073/pnas.1913980116. Available from: http://www.ncbi.nlm.nih.gov/pubmed/31636188. 10.1073/pnas.1913980116 PMC6883813 31636188

[4] Medecins Sans Frontieres (MSF). Crisis update - July 2019. DRC Ebola Outbreaks. 2019; Available from: https://www.msf.org/drc-ebola-outbreak-crisis-update. Accessed 10 Apr 2020.

[5] Maxmen A (2018). War zone complicates roll - out of Ebola vaccine. Nature..

[6] Vinck P, Pham PN, Bindu KK, Bedford J, Nilles EJ (2019). Institutional trust and misinformation in the response to the 2018–19 Ebola outbreak in North Kivu, DR Congo: a population-based survey. Lancet Infect Dis.

[7] Maxmen A (2019). Battling Ebola in a war zone. Nature..

[8] https://mailchi.mp/sante.gouv.cd/ebola_kivu_22juil19. Accessed 10 Apr 2020.

[9] Data DRC Ebola outbreak. Available from: https://docs.google.com/spreadsheets/d/e/2PACX-1vSrr9DRaC2fXzPdmOxLW-egSYtxmEp_RKoYGggt-zOKYXSx4RjPsM4EO19H7OJVX1esTtIoFvlKFWcn/pub?gid=1564028913&single=true&output=csv. [cited 2020 Mar 16].

[10] Sundberg R, Melander E. Introducing the UCDP Georeferenced Event Dataset. J Peace Res. 2013;50:523–32. 10.1177/0022343313484347.

[11] Eck K (2012). In data we trust? A comparison of UCDP GED and ACLED conflict events datasets. Coop Confl.

[12] Raleigh C, Linke A, Hegre H, Karlsen J (2010). Introducing ACLED: an armed conflict location and event dataset. J Peace Res.

[13] Democratic Republic of the Congo: high resolution population density maps. Available from: https://data.humdata.org/dataset/highresolutionpopulationdensitymaps-cod. Accessed 10 Apr 2020.

[14] Cori A, Ferguson NM, Fraser C, Cauchemez S (2013). Practice of epidemiology a new framework and software to estimate time-varying reproduction numbers during epidemics. Am J Epidemiol.

[15] WHO Ebola Response Team. Ebola virus disease in West Africa - the first 9 months of the epidemic and forward projections. N Engl J Med. 2014; Available from: http://www.ncbi.nlm.nih.gov/pubmed/25244186. [cited 2014 Sep 23]. 10.1056/NEJMoa1411100 PMC4235004 25244186

[16] R Core Team (2019). R: a language and environment for statistical computing.

[17] International Committee of the Red Cross. Yumbi, DRC: in the aftermath of violence. Available from: https://www.icrc.org/en/document/yumbi-drc-aftermath-violence. [cited 2020 Mar 20].

[18] Kraemer MUG, Reiner RC, Bhatt S. Causal inference in spatial mapping. Trends Parasitol. The Authors; 2019;35:743–746. 10.1016/j.pt.2019.06.005. Available from: https://linkinghub.elsevier.com/retrieve/pii/S1471492219301370. 10.1016/j.pt.2019.06.005 31279657

[19] Wagner Z, Heft-Neal S, Bhutta ZA, Black RE, Burke M, Bendavid E (2018). Armed conflict and child mortality in Africa: a geospatial analysis. Lancet.

[20] Abbara A, Rawson TM, Karah N, El-Amin W, Hatcher J, Tajaldin B (2018). Antimicrobial resistance in the context of the Syrian conflict: drivers before and after the onset of conflict and key recommendations. Int J Infect Dis.

[21] Epstein JM, Parker J, Cummings D, Hammond RA. Coupled contagion dynamics of fear and disease: mathematical and computational explorations. Galvani AP, editor. PLoS One 2008;3:e3955. doi: 10.1371/journal.pone.0003955. 10.1371/journal.pone.0003955 PMC2596968 19079607

[22] Kraemer MUG, Hay SI, Pigott DM, Smith DL, Wint GRW, Golding N (2016). Progress and challenges in infectious disease cartography. Trends Parasitol.

[23] Kraemer MUG, Faria NR, Nikolay B, Stasse S, RCR, Golding N (2016). Spread of yellow fever virus outbreak in Angola and the Democratic Republic of the Congo 2015–16: a modelling study. Lancet Infect Dis.

[24] Farrar JJ (2019). Stopping the gaps in epidemic preparedness. N Engl J Med.

[25] Pigott DM, et al. Local, national, and regional viral haemorrhagic fever pandemic potential in Africa: a multistage analysis. Lancet. 2017;390:2662–72. 10.1016/S0140-6736(17)32092-5 PMC5735217 29031848

[26] Mbala-Kingebeni P, Aziza A, Di Paola N, Wiley MR, Makiala-Mandanda S, Caviness K (2019). Medical countermeasures during the 2018 Ebola virus disease outbreak in the North Kivu and Ituri Provinces of the Democratic Republic of the Congo: a rapid genomic assessment. Lancet Infect Dis.

[27] Nuzzo JB, Inglesby TV. Ramping up the response to Ebola. N Engl J Med. 2018;379:2490–1. 10.1056/NEJMp1811988. 10.1056/NEJMp1814296 30485155

