# Supplementary material for: Dynamics of conflict during the Ebola outbreak in the Democratic Republic of the Congo 2018–2019
Source: BMC Med. 2020 Apr 27;18:113. doi: 10.1186/s12916-020-01574-1 (PMC7184697; doi:10.1186/s12916-020-01574-1)
Supplement: Supplementary file 1 — Additional file 1 : Figure S1. Histogram of the number of fatalities per conflict event using data from August 1, 2018 to July 26, 2019. Figure S2. Ratio of conflict events during the outbreak vs before the outbreak for health zones in Ituri and North Kivu that did not have transmission, those health zones with transmission and all other regions in the Democratic Republic of the Congo. Data come from two different sources (Uppsala and Armed Conflict Location & Event Data). Figure S3. a) Correlation of number of conflicts before the outbreak (1989 – July 31, 2018) and during the outbreak for each of the affected health zones. Gray line shows the regression line and shaded area is 95% confidence interval. b) Correlation between number fatalities before and after the conflict using the same time intervals than in a). Data are shown on the log scale. Gray line shows the regression line and shaded area is 95% confidence interval. Figure S4. Number of conflict events in the first half of the outbreak (August 2018 – January 2019) vs. second half of the outbreak (February 2019 – July 2019). Each dot represents one health zone in North Kivu and Ituri. Figure S5. Number of conflict events before the outbreak normalized by year (2017) and capita and during the outbreak normalized by year and capita. Figure S6. Number of conflicts per 10,000 vs. number of cases per 10,000 (Pearson’s r = 0.33, 95% CI: 0.05–0.57, p-value < 0.05). Blue dots represent areas that had reported Ebola transmission. Red dots represent locations that did not report transmission. Table S1. Coefficients of the linear univariate regression of the bi-weekly reproduction number (y) and conflict variables (x) (y = a + b*x). [file 12916_2020_1574_MOESM1_ESM.docx]

**Supplementary Appendix**

**Dynamics of conflict during the Ebola outbreak in the Democratic Republic of the Congo 2018-2019**

Moritz U. G. Kraemer^1,2,3,#^, David M. Pigott^4^, Sarah C. Hill^1^, Samantha Vanderslott^5^, Robert C. Reiner, Jr.^4^, Stephanie Stasse^6^, John S. Brownstein^2,3^, Bernardo Gutierrez^1,7^, Francis Dennig^8^, Simon I. Hay^4^, G.R. William Wint^9^, Oliver G. Pybus^1^, Marcia C. Castro^10^, Patrick Vinck^2,11,12^, Phuong N. Pham^2,11,12^, Eric J. Nilles^2,10,11^, Simon Cauchemez^13,#^

1. Department of Zoology, University of Oxford, United Kingdom
2. Harvard Medical School, Harvard University, United States
3. Computational Epidemiology Group, Boston Children’s Hospital, United States
4. Institute for Health Metrics and Evaluation, Department of Health Metrics Sciences, University of Washington, United States
5. Oxford Vaccine Group & Oxford Martin School, University of Oxford, Oxford, United Kingdom
6. European Union Delegation in Kinshasa, the Democratic Republic of the Congo
7. School of Biological and Environmental Sciences, Universidad San Francisco de Quito USFQ, Quito, Ecuador
8. Yale-NUS College, Singapore
9. Environmental Research Group Oxford, Department of Zoology, University of Oxford, United Kingdom
10. Harvard T.H. Chan School of Public Health, Boston United States
11. Program on Infectious Diseases and Emergencies, Harvard Humanitarian Initiative, Harvard University, United States
12. Brigham and Women’s Hospital, Boston, United States
13. Mathematical Modelling of Infectious Diseases Unit, Institut Pasteur, CNRS, UMR2000, France

Keywords: Ebola, conflict, violence, Democratic Republic of the Congo, outbreak

^#^Correspondence should be addressed to [moritz.kraemer@zoo.ox.ac.uk](mailto:moritz.kraemer@zoo.ox.ac.uk) and [simon.cauchemez@pasteur.fr](mailto:simon.cauchemez@pasteur.fr)

Supplementary Figures

Figure S1: Histogram of the number of fatalities per conflict event using data from August 1, 2018 to July 26, 2019.

Figure S2: Ratio of conflict events during the outbreak vs before the outbreak for health zones in Ituri and North Kivu that did not have transmission, those health zones with transmission and all other regions in the Democratic Republic of the Congo. Data come from two different sources (Uppsala and ACLED).


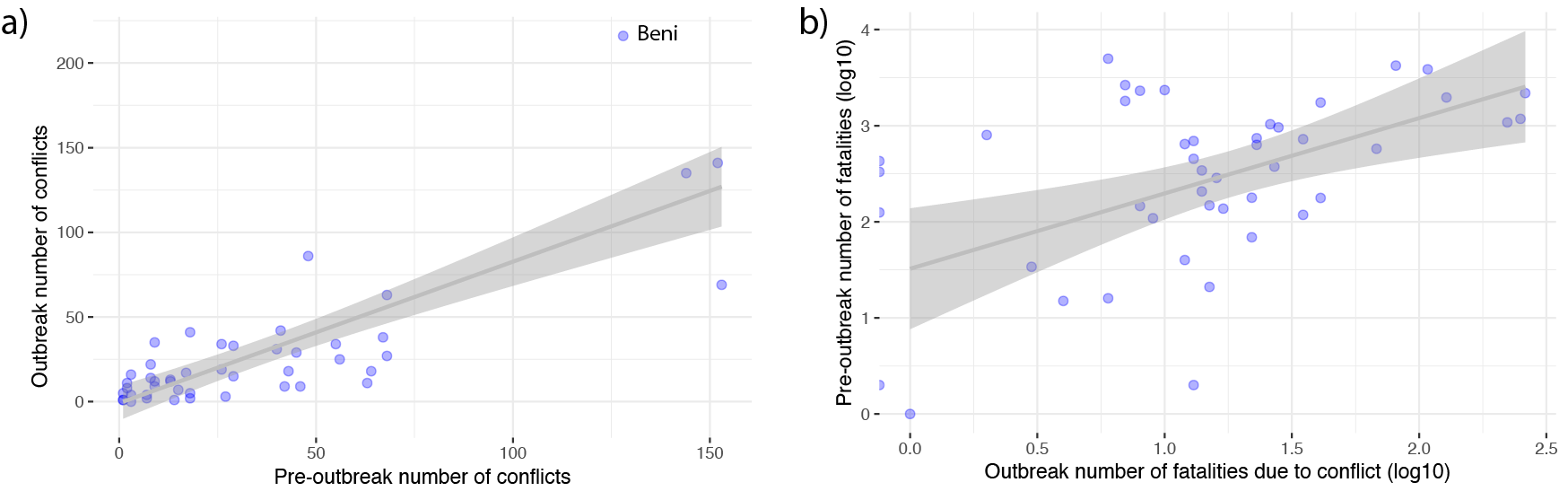


Figure S3: a) Correlation of number of conflicts before the outbreak (1989 – July 31, 2018) and during the outbreak for each of the affected health zones. Grey line shows the regression line and shaded area is 95% confidence interval. b) Correlation between number fatalities before and after the conflict using the same time intervals than in a). Data are shown on the log scale. Grey line shows the regression line and shaded area is 95% confidence interval.


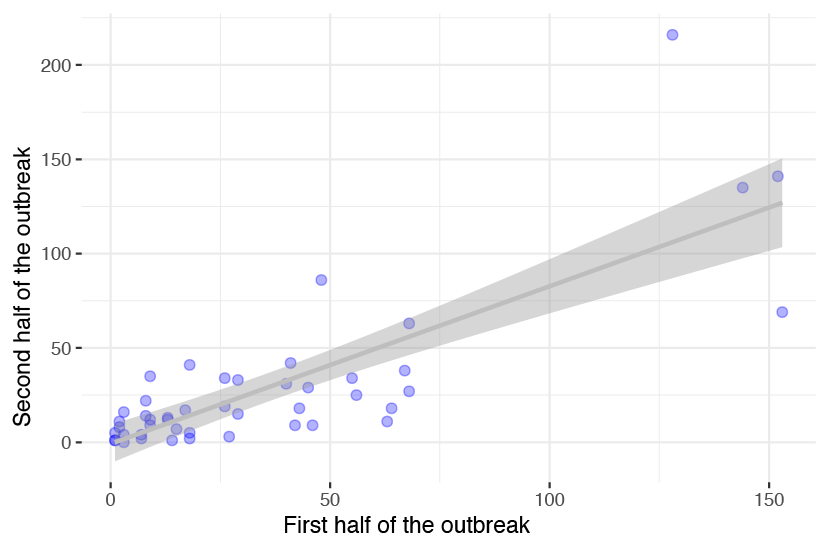


Figure S4: Number of conflict events in the first half of the outbreak (August 2018 – January 2019) vs. second half of the outbreak (February 2019 – July 2019). Each dot represents one health zone in North Kivu and Ituri.

Figure S5: Number of conflict events before the outbreak normalised by year (2017) and capita and during the outbreak normalised by year and capita.


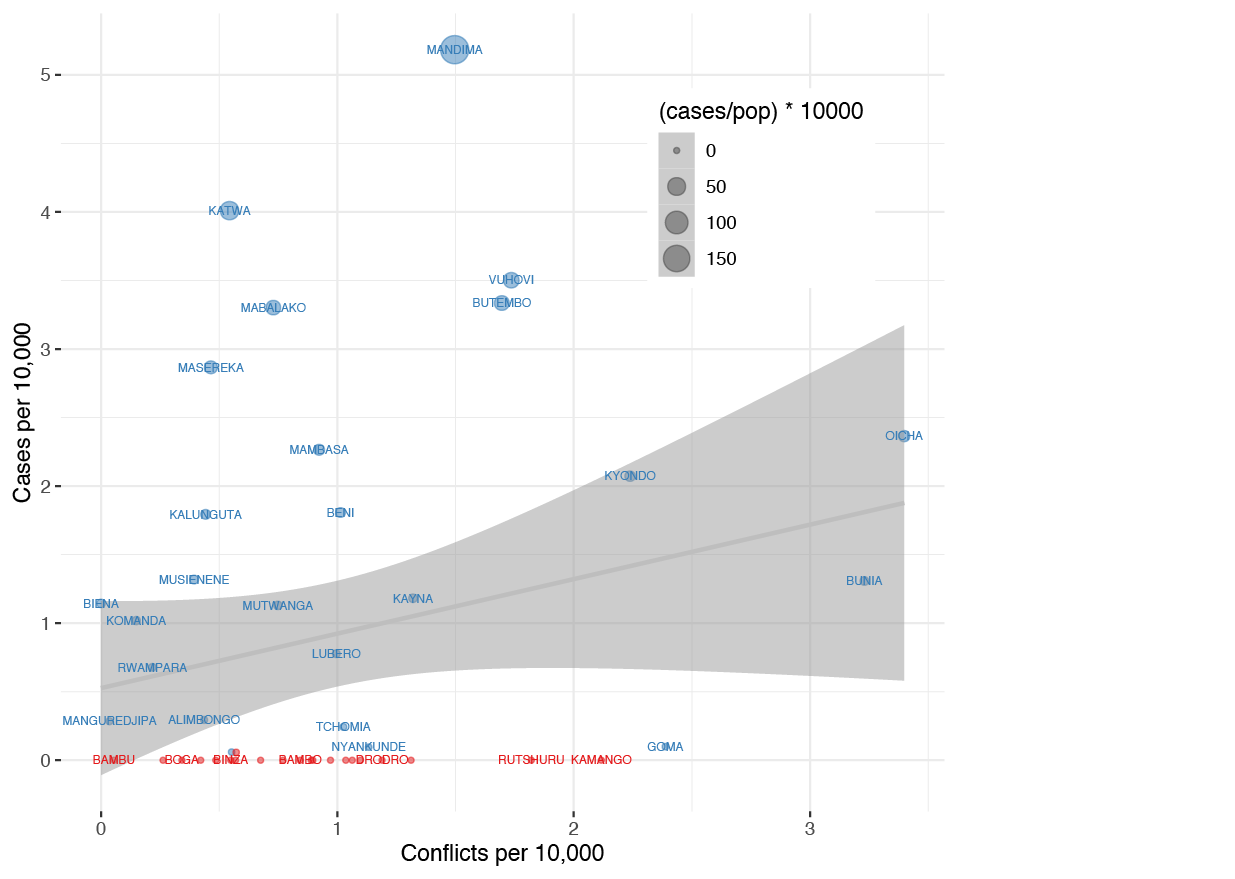


Figure S6: Number of conflicts per 10,000 vs. number of cases per 10,000 (Pearson’s r = 0.33, 95% CI: 0.05 – 0.57, p-value < 0.05). Blue dots represent areas that had reported Ebola transmission. Red dots represent locations that did not report transmission.

**Table S1:** Coefficients of the linear univariate regression of the bi-weekly reproduction number (y) and conflict variables (x) (y=a+b.x).

|  | a |  | b | p-value |
| --- | --- | --- | --- | --- |
| Number of conflicts |  |  |  |  |
| 0-week lag | 1.15 (0.08) | <0.0001 | 0.03 (0.03) | 0.36 |
| 2-week lag | 1.16 (0.09) | <0.0001 | 0.02 (0.03) | 0.49 |
| 4-week lag | 1.16 (0.08) | <0.0001 | 0.03 (0.03) | 0.43 |
| Number of battles |  |  |  |  |
| 0-week lag | 1.14 (0.08) | <0.0001 | 0.09 (0.06) | 0.13 |
| 2-week lag | 1.18 (0.08) | <0.0001 | 0.02 (0.06) | 0.81 |
| 4-week lag | 1.17 (0.08) | <0.0001 | 0.03 (0.06) | 0.59 |
| Number of violence t. civilians |  |  |  |  |
| 0-week lag | 1.19 (0.08) | <0.0001 | 0.01 (0.10) | 0.92 |
| 2-week lag | 1.12 (0.08) | <0.0001 | 0.15 (0.08) | 0.08 |
| 4-week lag | 1.15 (0.08) | <0.0001 | 0.09 (0.08) | 0.27 |
| Number of deaths |  |  |  |  |
| 0-week lag | 1.17 (0.08) | <0.0001 | 0.007 (0.012) | 0.56 |
| 2-week lag | 1.17 (0.08) | <0.0001 | 0.006 (0.012) | 0.62 |
| 4-week lag | 1.18 (0.08) | <0.0001 | 0.002 (0.012) | 0.87 |
